# Response and prognosis to neoadjuvant chemotherapy in women early breast cancer of HER2-low status

**DOI:** 10.3389/fonc.2025.1596156

**Published:** 2025-06-09

**Authors:** Yongtao Li, Tangnuer Buerliesi, Wenting Xu, Lina Yi, Fulati Wuwalihan

**Affiliations:** Department of Breast Surgery, Xinjiang Medical University Affiliated Tumor Hospital, Urumqi, China

**Keywords:** breast cancer, neoadjuvant chemotherapy, HER2-low expression, pathological complete response, prognostic analysis

## Abstract

**Objective:**

With the significant clinical benefits of antibody-coupled drugs (ADCs) against HER2, the HER2-low-expressing population has come into focus. HER2-low-expressing patients express this membrane protein despite the absence of HER-2 amplification. Whether there is a difference in outcome and prognosis between patients with HER2-low expression and those with HER2–0 expression has not yet been clarified, and more clinical data are needed to characterize them.

**Methods:**

Clinical and pathological data of HER2-low versus HER2-0-expressing breast cancer patients treated with neoadjuvant chemotherapy (NACT) and operated at Xinjiang Uygur Autonomous Region Cancer Center from 2015 to 2018 were reviewed, and the patients were analyzed and studied in terms of pathologic complete response (PCR), overall survival (OS) and disease-free survival (DFS).

**Results:**

A total of 283 breast cancer patients were included, 102 (36.04%) with HER2–0 expression and 181 (63.96%) with HER2-low expression, with clinical stage II-III. After the study, the pCR rates of HER2–0 and HER2-low tumors were found to be 19.61% and 11.05%, respectively, which were not statistically different (*p*=0.071); there were also no significant differences in the pCR rates in both hormone receptor-positive (HR+) and negative (HR-) subgroups of patients. DFS and OS were analyzed for all patients, and there was no statistically significant difference in DFS (*p*=0.16) and OS (*p*=0.33) between HER2–0 and HER2-low cases; however, in the HR- subgroup, DFS was worse in HER2-low patients (*p*=0.027), yet there was no statistically significant difference in OS (*p*=0.24); in the HR+ subgroup, the HER2 status was not associated with DFS and OS. We also analyzed DFS and OS in PCR and nonPCR patients, and there was no statistically significant difference in DFS (*p*=0.29) and OS (*p*=0.54) between HER2–0 and HER2-low cases in PCR patients, and no difference in DFS and OS between HER2–0 and HER2-low cases in nonPCR patients.

**Conclusion:**

In breast cancer patients receiving neoadjuvant chemotherapy, no significant differences in chemosensitivity or prognostic outcomes were observed between HER2-low and HER2–0 tumors, considering HR expression subtypes and other current clinicopathological features.

## 1 Introduction

Breast cancer has become the malignant tumor with the highest global incidence rate and the leading cause of cancer deaths in women worldwide, and China has the highest percentage of new cases of breast cancer in the world, seriously endangering the lives and health of women (1). Treatment decisions for breast cancer are usually based on traditional histopathologic findings (2), and clinically breast cancer is divided into four main subtypes with different prognoses: Luminal A, Luminal B, human epidermal growth factor receptor 2 (HER2,also known as ERBB2)-positive, and triple-negative breast cancer (TNBC) (3). HER2 is an important driver gene and prognostic indicator of breast cancer, and is also a major predictor of the efficacy of anti-HER2 drug therapy (4). In HER2-positive breast cancer, ERBB2 gene amplification leads to HER2 overexpression, and patients without anti-HER2 treatment have more aggressive tumors and poorer patient prognosis (5). Currently, multiple targeted drugs against HER2 have significantly improved the clinical prognosis of early and advanced HER2-positive breast cancer (4). In breast cancer, about 45%-55% showed low HER2 expression, i.e., immunohistochemistry (IHC) 1+, or IHC 2+ and no amplification of the HER2 gene by *in situ* hybridization (ISH) (6). In recent years, the treatment of HER2-low expression breast cancer has become a hot issue in breast cancer diagnosis and treatment. With the establishment of the efficacy of antibody-drug conjugate (ADC) in HER2-low expression breast cancer patients, HER2-low expression may become a new targeted therapeutic subtype of breast cancer, and the novel ADC has become a new therapeutic option for HER2- low expression advanced breast cancer patients (7). HER2-low breast cancer represents a clinically distinct subgroup with unique biological behaviors that influence responses to neoadjuvant chemotherapy. Recent advances highlight the critical role of tumor microenvironment (TME) components in modulating chemosensitivity. The hypoxic state and chronic inflammatory response of the TME have been proven to be associated with chemotherapy resistance. In HER2- low breast cancer, the dense extracellular matrix may limit drug penetration through its physical barrier effect and weaken the efficacy of chemotherapy by recruiting immunosuppressive cells such as M2-type TAMs (8);Yanni Xu et al. (9)utilized targeted CD206 imaging to reveal spatial heterogeneity in M2-polarized TAMs within HER2-low tumors, which correlate with reduced chemotherapy efficacy through immunosuppressive cytokine secretion (e.g., IL-10, TGF-β). This is similar to the conclusion of another study (10). Some scholars (11) demonstrated that non-invasive magnetic stimulation disrupts F-actin cytoskeletal dynamics, enhancing anthracycline penetration in HER2-low models by increasing vascular permeability;Ji Xinmiao et al. (12) also mentioned that magnetic field perturbation inhibits the metastasis of breast cancer, which may be related to chemotherapy sensitivity. Studies by Asma Mokashi et al. identified progesterone receptor (PR)-mediated ERK/MAPK pathway activation as a driver of chemoresistance in HER2-low tumors using network pharmacology, emphasizing the interplay between HR status and HER2 biology (13). This study aims to determine whether HER2-low status independently predicts chemosensitivity or survival in NAC-treated patients, beyond HR subtypes. Clarifying HER2-low for chemotherapy sensitivity research can promote the development of precision medicine and provide ideas for more personalized development and use of new drugs. Pathologic complete response (pCR) is widely used to assess the sensitivity of chemotherapy in breast cancer, and achieving pCR after neoadjuvant chemotherapy is associated with significantly improved EFS and OS (14). Currently, a variety of innovative methods can assess the efficacy and prognosis of neoadjuvant chemotherapy in real time.Haonan Xu et al. (15) developed Cu-MOF nanoparticles for dynamic MRI monitoring of tumor vascular normalization, where changes in the transfer constant predict pCR. Furthermore, some scholars (16) have linked PTBP2-mediated alternative splicing of IRF9 to TAM repolarization, with elevated PTBP2 expression predicting inferior DFS in HER2-low cohorts. In this study we evaluated the prognostic and predictive value of HER2-low status in breast cancer patients receiving neoadjuvant chemotherapy (17).

## 2 Patients and methods

### 2.1 Patient data

We conducted a retrospective analysis of breast cancer (BC) patients who visited the Xinjiang Uygur Autonomous Region Cancer Center for neoadjuvant chemotherapy and surgery between 2015 and 2018. Our inclusion criteria were as follows: age ≥18 years, pre-treatment biopsy consistent with invasive breast cancer, HER2 IHC 0, 1+, or 2+/ISH-, surgical treatment after receiving NACT, and hormone receptor could be positive or negative. Patients with HER2-positive breast cancer (IHC 3+ or IHC 2+/ISH+) or HER2 IHC 2+/ISH-equivocal, clinical IV, bilateral or previous history of invasive breast cancer or history of other primary tumors, patients receiving radiotherapy or hormonal therapy alone were excluded.

The local institutional internal ethical review board approved the study. Age at diagnosis, menopausal status, HR, Ki67, HER2 status, grading, histologic type, preoperative pathological N-stage, postoperative pathological N-stage, clinical stage at diagnosis, pAJCC, chemotherapy, surgery, ORR, pCR, DFS, OS, and tumor efficacy evaluations (CR, PR, SD, and PD) were collected from the patients’ medical records, pathology reports, and follow-up examinations. When it comes to neoadjuvant chemotherapy regimens, there are mainly three types. First, the anthracycline-taxane class, which includes AC-T, namely, doxorubicin and cyclophosphamide for 4 cycles, followed by paclitaxel administered 12 times or docetaxel administered 4 times; and TAC, that is, docetaxel, doxorubicin, and cyclophosphamide administered 6 times. Second, the platinum-containing class. There is TCbH, that is, paclitaxel and carboplatin administered 6 times plus trastuzumab, which is used for HER2-positive patients; and there is also EC-D, namely, epirubicin and cyclophosphamide administered 4 times, followed by docetaxel and cisplatin administered 4 times. Third, other classes. For example, CMF, which is composed of cyclophosphamide, methotrexate, and 5-fluorouracil, requires 6 courses of treatment and is often used for elderly patients or those with comorbidities. In our study, we collectively refer to the latter two as “others”, that is, the neoadjuvant chemotherapy regimens are classified and studied into two categories: anthracycline combined with paclitaxel and others. Pathologic complete response (pCR) was defined as ypT0/is and ypN0 on surgical specimens after neoadjuvant chemotherapy. Time from pathologic diagnosis to death from any cause was defined as overall survival (OS). The time from surgery to disease recurrence, metastasis, or death from any cause was defined as disease-free survival (DFS). Time to pathological diagnosis, surgery, recurrence, and metastasis were collected to calculate DFS and OS. Solid Tumor Response Evaluation Criteria (RECIST 1.1) were used to assess tumor response after NAC (18).

### 2.2 Immunohistochemical evaluation

All patients included in this study underwent diagnostic and immunohistochemical testing for breast cancer at our institution, and these evaluations were performed prior to any treatment of the biopsy specimens. HER2 status was determined based on the pathology report derived from diagnostic biopsy analysis. HER2 was assessed using standard antibodies and FISH techniques, diagnosis was determined using ASCO/CAP guidelines for HER2 status: HER2-low was defined as IHC 1+ or IHC 2+/FISH non-amplified, and HER2–0 was defined as IHC 0 (17). Regarding tumor hormone receptor (HR) status, tumors were defined as HR-positive (HR+) if estrogen or progesterone receptors were expressed in >1% of tumor cells, and HR-negative (HR-) for the rest.

### 2.3 Statistical analysis

Clinicopathologic characteristics were expressed as descriptive statistics, such as patient means, standard deviations, or percentages. Percentages were calculated from complete data. Pearson χ2 or Fisher exact tests were used to compare the differences between the HER2-low and HER2–0 groups. To investigate the differences, continuous variables were compared between the two groups using the t-test in case the variables were normally distributed and the Wilcoxon test for non-normal distribution. Categorical variables were compared between groups using the chi-square test. The Kaplan-Meier method was used to plot the survival curves, and log-rank analysis was utilized to compare the OS, DFS. Disease-free survival (DFS) was defined as the number of months between the date of breast cancer diagnosis and the first recurrence or metastasis, or death. Overall survival (OS) was defined as the time in months between breast cancer diagnosis and death from any cause or last follow-up. Cox univariate and proportional risk multivariate regression was used to identify independent predictors of survival. Statistical tests were bilateral with a significance threshold fixed at 5%.

## 3 Results

### 3.1 pCR rates by HER2 status

**Figure 1:**
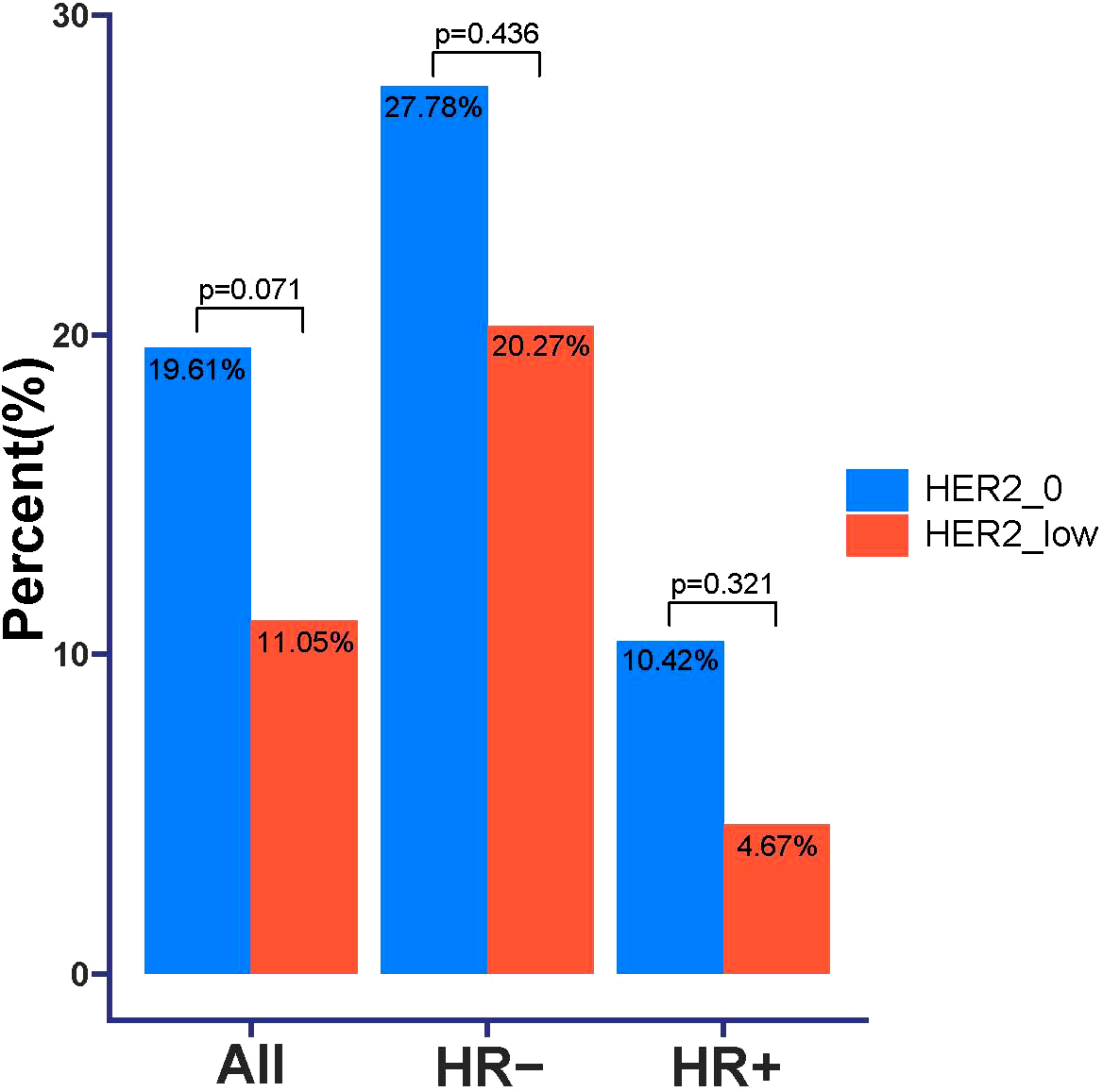
Comparison of pathologic complete response (pCR) in HER2–0 and HER2-low breast cancer patients.

According to the inclusion and exclusion criteria, a total of 283 patients with HER2 unamplified breast cancer were finally included in this study. Among all these patients, 102 patients (36.04%) had HER2–0 expression and 181 patients (63.96%) had HER2-low expression. We divided the patients into 155 (54.8%) in the HR+ group with a mean age at diagnosis of 46.7 years and 128 (45.2%) in the HR- group with a mean age at diagnosis of 48.3 years based on HR status. The proportions of HER2–0 and HER2-low were not the same between HR+ and HR- breast cancers, and in the HR+ tumors HER2-low in 107 cases (69.03%) and 74 cases (57.81%) in HR- tumors. **Table 1** shows the complete baseline demographic and clinicopathologic characteristics of all patients. When comparing HER2-low and HER2–0 tumors in HR+ and HR- tumors, respectively, we found that there was no significant difference in clinicopathological characteristics between the two groups. Pathologic complete response was achieved in 40 of all patients (14.13%), with pCR rates of 19.61% and 11.05% for HER2–0 and HER2-low tumors, respectively, which were not statistically different (*p*=0.071). In the HR- tumor subgroup, the pCR rate showed higher, but the pCR rates for HER2–0 and HER2-low tumors were 27.78% and 20.27%, respectively, with no statistical difference (*p*=0.436), and in the HR+ tumor subgroup, the pCR rates for HER2–0 and HER2-low tumors were 10.42% and 4.67%, respectively, as well as no statistical difference (*p*=0.321). This result also suggests that HER2 status (low/0) is not associated with the probability of achieving a significant difference in pCR in either HR+ or HR- breast cancers (**Figure 1**).

**Table 1:** Baseline clinical and pathological characteristics of the patients.

Characteristic	HR+ (N=155)		*p* value	HR- (N=128)		*p* value
	HER2 0 (N=48)	HER2 low (N=107)		HER2 0 (N=54)	HER2 low (N=74)	
Age, years, mean ± sd	46.5 ±7.53	46.8±7.01	0.867	48.6±6.65	48.1 ±7.93	0.679
Ki67, %, mean ± sd	37.1 ±17.6	38.6 ±16.5	0.625	43.2 ±16.7	42.6 ±17.0	0.841
Menopausal status (%)			0.671			0.408
Premenopausal	35 (72.9%)	83 (77.6%)		38 (70.4%)	$8 (78.4%)	
Postmenopausal	13 (27.1%)	24 (22.4%)		16 (29.6%)	16 (21.6%)	
Histological type (%)			0.327			0.892
Ductal	45 (93.8%)	93 (86.9%)		48 (88.9%)	64 (86.5%)	
Other	3 (6.25%)	14 (13.1%)		6 (11.1%)	10 (13.5%)	
cN stage (%)			0.245			0.346
NO	6 (12.5%)	21 (19.6%)		2 (3.70%)	8 (10.8%)	
N1	26 (54.2%)	58 (54.2%)		35 (64.8%)	47 (63.5%)	
N2	3 (27.1%)	16 (15.0%)		14 (25.9%)	13 (17.6%)	
N3	3 (6.25%)	12 (11.2%)		3 (5.56%)	6 (8.11%)	
Clinical stage at diagnosis (%)			0.332			1.000
II	33 (68.8%)	83 (77.6%)		33 (61.1%)	46 (62.2%)	
III	15 (31.2%)	24 (22.4%)		21 (38.9%)	28 (37.8%)	
Grade (%)			0.123			0.813
II	40 (83.3%)	75 (70.1%)		39 (72.2%)	56 (75.7%)	
III	8 (16.7%)	32 (29.9%)		15 (27.8%)	18 (24.3%)	
Chemotherapy (%)			1.000			0.983
Anthracycline + taxane	42 (87.5%)	93 (86.9%)		44 (81.5%)	59 (79.7%)	
Other	6 (12.5%)	14 (13.1%)		10 (18.5%)	15 (20.3%)	
Breast surgery (%)			0.192			1.000
Mastectomy	42 (87.5%)	101 (94.4%)		43 (79.6%)	60 (81.1%)	
Breast conserving	6 (12.5%)	6 (5.61%)		11 (20.4%)	14 (18.9%)	
ypN stage (%)			0.167			0.706
NO	36 (75.0%)	67 (62.6%)		34 (63.0%)	49 (66.2%)	
N1	12 (25.0%)	0 (28.0%)		17 (31.5%)	(28.4%)	
N2	0 (0.00%)	7 (6.54%)		2 (3.70%)	(5.41%)	
N3	0 (0.00%)	3 (2.80%)		(1.85%)	(0.00%)	
ypTNM (%)			0.196			0.088
0	4 (8.33%)	5 (4.67%)		15 (27.8%)	15 (20.3%)	
I	2 (66.7%)	(57.9%)		19 (35.2%)	37 (50.0%)	
II	12 (25.0%)	33 (30.8%)		9 (35.2%)	16 (21.6%)	
III	0 (0.00%)	7 (6.54%)		1 (1.85%)	6 (8.11%)	

### 3.2 Survival analysis in HR subgroups

We next examined the long-term outcomes, including DFS and OS, of a cohort of breast cancer patients treated with NAC to investigate the impact of HER2-low expression status on patient survival. The median follow-up for the entire cohort was 5.75 years. By the end of follow-up, the incidence of DFS was 84/283 (29.6%) and the incidence of OS events was 47/283 (16.6%).

**Figure 2:**
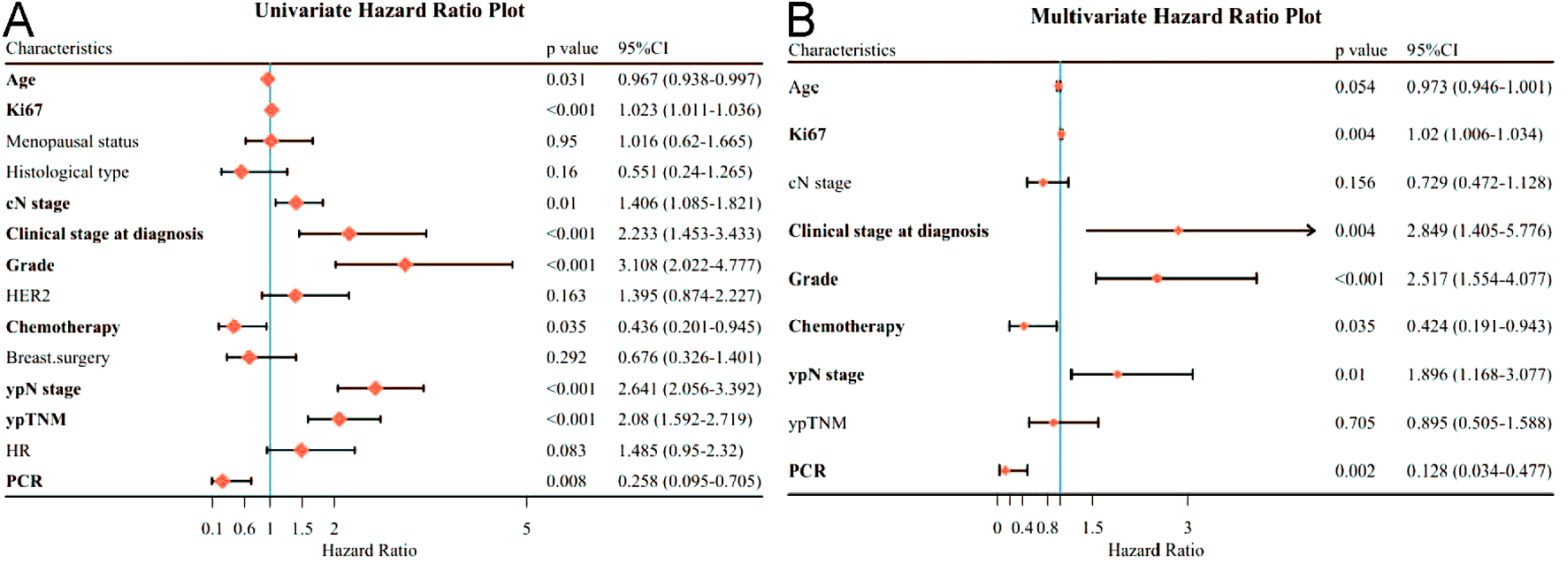
Forest plot affecting patients’ disease-free survival (DFS). **(A)** Results of unifactorial analysis affecting DFS; **(B)** Results of multifactorial analysis affecting DFS.

In these patients, univariate analysis showed that clinicopathological factors associated with worse DFS were age (*p*=0.031), Ki67 expression (*p*<0.001), higher cN stage (*p*=0.01), higher Clinical stage at diagnosis (*p*<0.001), higher tumor grade (*p*<0.001), and higher tumor grade (*p*<0.001), Chemotherapy (*p*=0.035), ypN stage (*p*<0.001) and ypTNM (*p*<0.001) and lower PCR (*p*=0.008) (**Figure 2A**). By multifactorial analysis, Ki67 expression (*p*=0.004), Clinical stage at diagnosis (*p*=0.004), Grade (*p*<0.001), Chemotherapy (*p*=0.035), ypN stage (*p*=0.01) and PCR (*p*=0.002) were associated with poorer DFS independently (**Figure 2B**).

**Figure 3:**
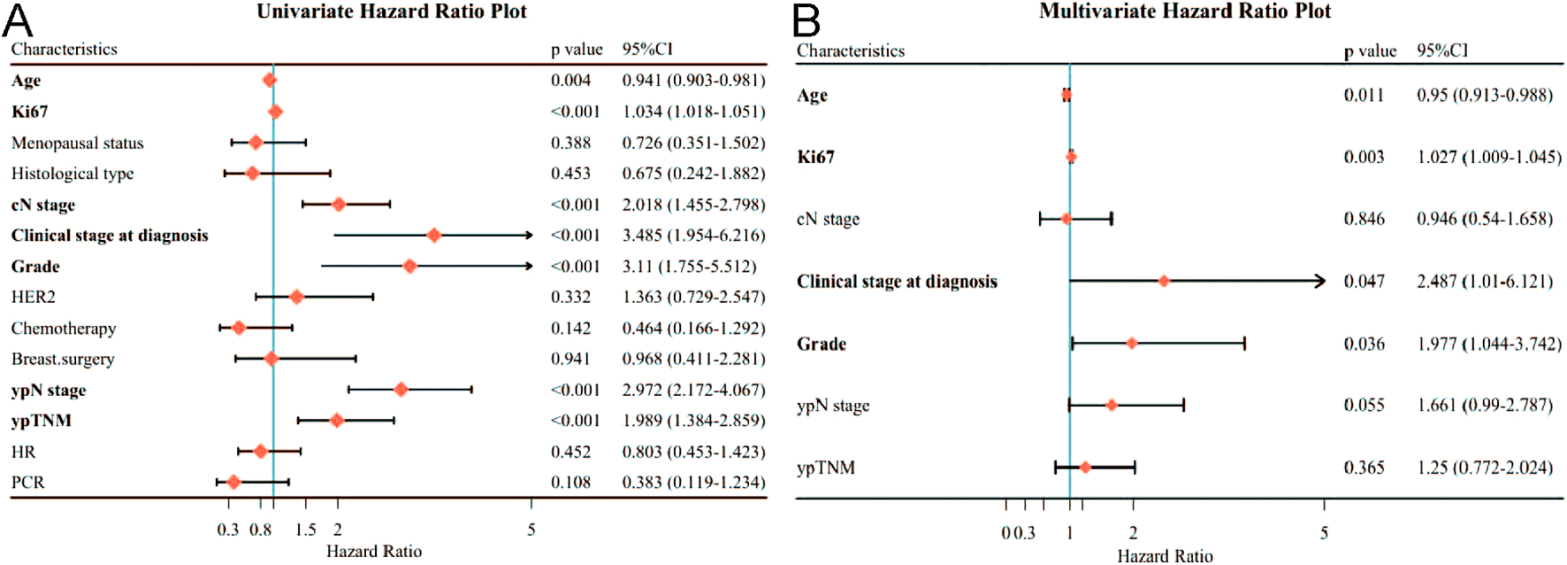
Forest plot affecting patients’ OS. **(A)** Results of unifactorial analysis affecting OS; **(B)** Results of multifactorial analysis affecting OS.

Regarding OS in patients receiving neoadjuvant therapy, by univariate analysis, only AGE (*p*=0.004), Ki67 expression (*p*<0.001), higher cN stage (*p*<0.001), higher Clinical stage at diagnosis (*p*<0.001), higher Grade (*p*<0.001), higher ypN stage (*p*< 0.001) and ypTNM (*p*< 0.001) were associated with OS (**Figure 3A**). By multivariate analysis, age (*P*=0.011), Ki67 expression (*P*=0.003), clinical stage at diagnosis (*P*=0.047), and Grade (*P*=0.036) remained independently associated with poorer OS (**Figure 3B**).

### 3.3 The impact of HER2 status on survival outcomes

**Figure 4:**
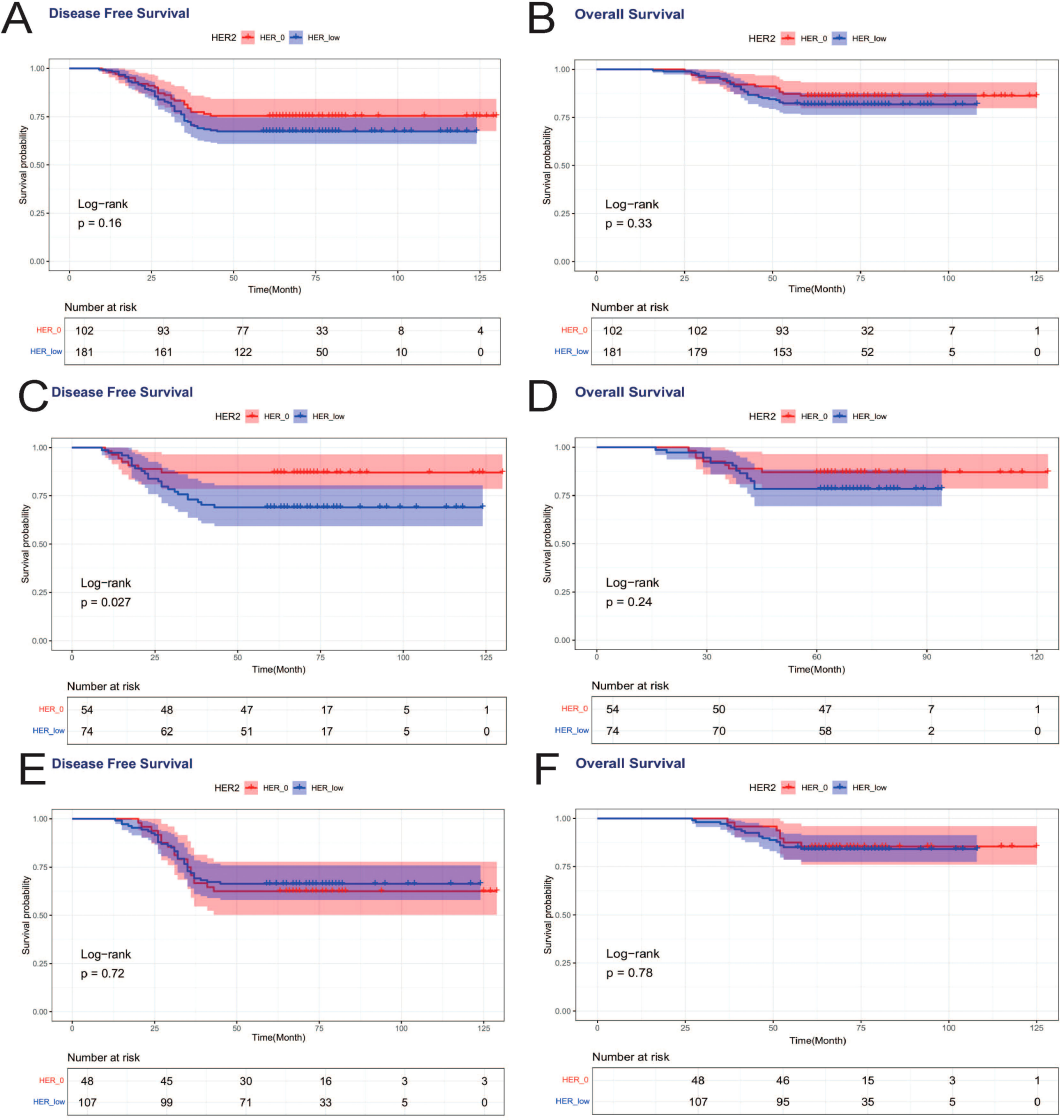
Effects of HER2-low and HER2–0 on patients’ DFS and OS. **(A)** DFS effect on all patients; **(B)** OS effect on all patients; **(C)** DFS effect on HR- patients; **(D)** OS effect on HR- patients; **(E)** DFS effect on HR+ patients; **(F)** OS effect on HR+ patients.

When DFS and OS were analyzed for all patients, HER2 status was not associated with either DFS or OS, and there was no statistically significant difference between the DFS curves (*p*=0.16) and OS curves (*p*=0.33) for HER2–0 and HER2-low cases (**Figure 4A**). We again analyzed in HR+ and HR- subgroups, and in HR-breast tumors, HER2 status affected patients’ DFS, and the DFS curves of HER2-low patients were worse than those of HER2–0 patients, which was statistically different (*p*=0.027). However, HER2 status was not associated with OS in HR-breast patients (**Figure 4B**). In HR+ breast tumors, HER2 status was not associated with either DFS or OS (**Figure 4C**).

**Figure 5:**
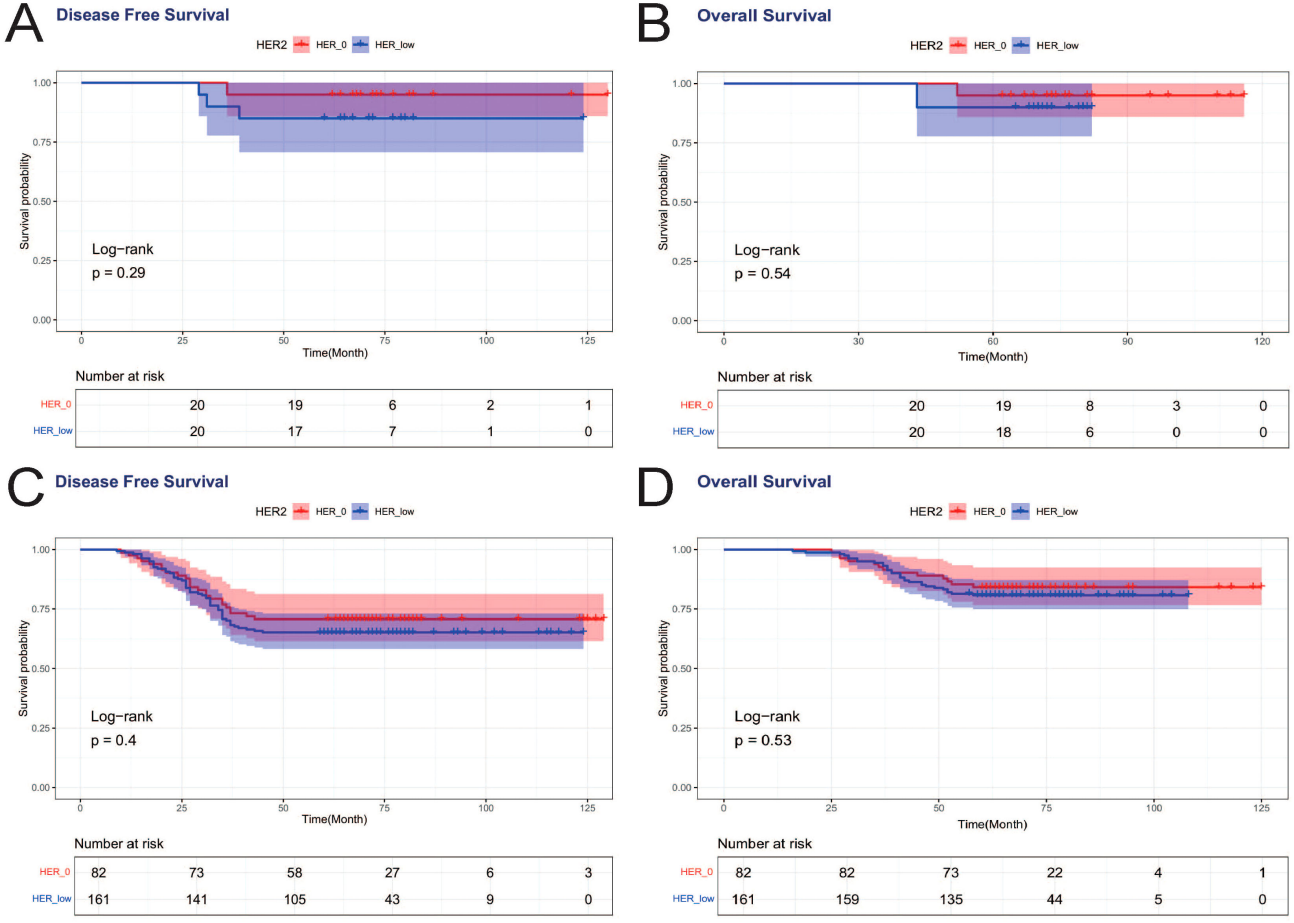
DFS and OS effects of HER2-low and HER2–0 on PCR vs. nonPCR patients. **(A)** DFS effects on PCR patients; **(B)** OS effects on PCR patients; **(C)** DFS effects on nonPCR patients; **(D)** OS effects on nonPCR patients.

Achieving pCR was associated with improved DFS, with a statistically significant difference in DFS curves between PCR and nonPCR cases (*p*=0.008) (**Figure 2A**), and PCR patients also showed a trend toward better OS, but not statistically different (*p*=0.108) (**Figure 3A**). Therefore, we again compared the DFS and OS effects of HER2-low and HER2–0 in PCR vs. nonPCR patients. In PCR patients, DFS curves (*p*=0.29) and OS curves (*p*=0.54) were not statistically different between HER2–0 and HER2-low cases (**Figures 5A, B**). In nonPCR patients, the DFS and OS curves of HER2–0 and HER2-low cases were also not statistically different (**Figures 5C, D**).

## 4 Discussion

Currently, the most widely used clinical staging of breast cancer is based on comparing their clinicopathological, genetic and immunohistochemical results, that is, staging from their pathological, genetic and immunological characteristics, in order to provide more personalized treatment plans and more precise medical treatment. Studies have found that more than half of HER2-negative patients have low HER2 expression (IHC 1+ or IHC 2+/ISH negative), and are considered to be incapable of receiving anti-HER2 therapy despite the presence of HER2 expression (17, 19). It was discovered in the study that HER2-low might be able to shake up the existing molecular typing of HER2-positive versus HER2-negative breast cancers due to the unique pathology it exhibits. HER2-low breast cancer was proposed as a new clinical type after phase I clinical studies first demonstrated the efficacy of new antibody-drug couplings in tumors with weak expression of the HER2 protein (20, 21). In 2022, the results of the DESTINY-Breast04 study, which enrolled patients with HER2-overexpressing advanced breast cancer (mBC), were published and found that all patients receiving T-DXd treatment had significantly longer PFS and OS, and rapidly changed international authoritative guidelines such as NCCN and ESMO in a short period of time, and was approved as the first HER2-targeted therapy for patients with HER2-low mBC (22). This has led to a strong interest in the specific subtype of HER2-low, as well as more opportunities for the future treatment of this subtype.

In our study, we found that HER2-low expression was more common in HR+ tumors (HR+ group: 69.03%, HR- group: 57.81%), a result that seems to be basically similar to previous studies (19), i.e., HR hormone receptors tend to be more expressed in HER2-low expressing breast cancers. This result was reached in a large study: HER2-low in the HR+ group showed higher OS and DFS at 60 months; and the following conclusions were obtained: HER2-low breast cancers showed less aggressive clinicopathologic features compared to HER2–0 cases; and the prognostic impact of HER2-low on resectable breast cancers varied depending on the patient’s HR expression status (23). Therefore, considering HR+ and HR- as confounding factors that may affect our study of HER2-low versus HER2–0 tumors, we divided these 283 breast cancer patients into HR+ and HR- subgroups for analysis. Previous studies have shown that HER2-amplified breast cancers receiving neoadjuvant chemotherapy are able to achieve higher pCR rates than HER2-non-amplified breast cancers (24). So does HER2–0 expression versus HER2-low expression affect the PCR rate of neoadjuvant chemotherapy in breast cancer? Our results found that the pCR rates of HER2–0 and HER2-low tumors were 19.61% and 11.05%, respectively, with no statistical difference. The PCR rate was higher in HR- patients, but there was no significant difference in PCR rates between HER2–0 and HER2-low patients in either HR+ or HR- breast cancer subgroups. We found low PCR rates in HR+ tumors and no difference in PCR rates between HER2-low and HER2–0 tumors, and the results do not support an association between these two expression states of HER2-negativity and pCR in patients. In HR-breast cancer, related studies reported that the difference in pCR incidence between HER2–0 and HER2-low tumors was not statistically significant (25–29), which is consistent with our findings. Research has revealed that HER2-low tumors exhibit unique biological characteristics that enable them to achieve different survival and prognosis from HER2–0 tumors after neoadjuvant chemotherapy (30, 31). According to our study, the two statuses of HER2-low or HER2–0 did not seem to have different clinicopathological features in breast cancer, and both expression statuses had no effect on the pCR as well as DFS and OS of the patients, although in the HR- subgroup of patients, HER2–0 seemed to have a better DFS compared to the HER2-low tumors (*P*=0.027), taking into account the fact that in the association between pCR and survival in HR- patients, we get that this conclusion does not contradict the existing logic (32). And although OS also showed this trend, there was no statistical difference, which was considered to be related to the small sample size of our study. This also suggests that for HER2-low expression patients, we should put them in traditional molecular typing, and if the patients are hormone receptor positive HER2-low expression, they should be regarded as hormone receptor positive Luminal type patients; while if the patients are hormone receptor negative HER2-low expression, they should be regarded as triple negative patients. In other words, traditional molecular typing is still of key, important significance for treatment. A most recent research has reached a comparable conclusion as well (33).

Therefore, the significance and value of neoadjuvant chemotherapy in HER2-low-expressing breast cancers still needs to be further explored, and it is unclear whether this population can receive the maximum benefit from neoadjuvant chemotherapy (34). HER2-low expression has received much attention because of the DB04 clinical study, the future DB06 clinical study, and the many novel anti-HER2 ADC agents that have been carried out. The outcome and expected outcome of the treatment have led to a strong interest in this specific subtype, as well as more opportunities for the future treatment of this subtype. HER2-low tumors have a specific biology and show some differences in response to treatment and prognosis, which is crucial in the treatment of drug-resistant HR-breast cancers. Our findings could provide an update of breast cancer subtypes and improve future diagnostic and therapeutic strategies.

## 5 Conclusion

Breast cancer patients with HER2-low and HER2–0 did not differ significantly in terms of near-term efficacy (pCR rate) and long-term prognosis (DFS and OS) after neoadjuvant chemotherapy, although patients showing HER2-low in the HR-subgroup of patients may have worse DFS. Combined with the current study reports, HER2-low is accepted by the majority of the population as a therapeutic subtype in breast cancer treatment, but whether HER2-low can be considered a new molecular subtype is still controversial and still needs to be proved by more and larger studies.

## Data Availability

The raw data supporting the conclusions of this article will be made available by the authors, without undue reservation.
